# CXCR4 Inhibition Enhances Efficacy of FLT3 Inhibitors in FLT3-Mutated AML Augmented by Suppressed TGF-β Signaling

**DOI:** 10.3390/cancers12071737

**Published:** 2020-06-30

**Authors:** Bo-Reum Kim, Seung-Hyun Jung, A-Reum Han, Gyeongsin Park, Hee-Je Kim, Bin Yuan, Venkata Lokesh Battula, Michael Andreeff, Marina Konopleva, Yeun-Jun Chung, Byung-Sik Cho

**Affiliations:** 1 Leukemia Research Institute, College of Medicine, The Catholic University of Korea, Seoul 06591, Korea; coolperson@catholic.ac.kr (B.-R.K.); areum0810@naver.com (A.-R.H.); cumckim@catholic.ac.kr (H.-J.K.); 2 Department of Biochemistry, College of Medicine, The Catholic University of Korea, Seoul 06591, Korea; hyun@catholic.ac.kr; 3 Department of Cancer Evolution Research Center, College of Medicine, The Catholic University of Korea, Seoul 06591, Korea; 4 Department of Pathology, College of Medicine, College of Medicine, The Catholic University of Korea, Seoul 06591, Korea; gspark@catholic.ac.kr; 5 Department of Hematology, Catholic Hematology Hospital, Seoul St. Mary’s Hospital, College of Medicine, The Catholic University of Korea, Seoul 06591, Korea; 6 Section of Molecular Hematology and Therapy, Department of Leukemia, The University of Texas MD Anderson Cancer Center, Houston, TX 77030, USA; BYuan@mdanderson.org (B.Y.); vbattula@mdanderson.org (V.L.B.); mandreef@mdanderson.org (M.A.); 7 Department of Leukemia, the University of Texas MD Anderson Cancer Center, Houston, TX 77030, USA; mkonople@mdanderson.org

**Keywords:** CXCR4, FLT3-ITD, acute myeloid leukemia, LY2510924, quizartinib

## Abstract

Given the proven importance of the CXCL12/CXCR4 axis in the stroma–acute myeloid leukemia (AML) interactions and the rapid emergence of resistance to FLT3 inhibitors, we investigated the efficacy and safety of a novel CXCR4 inhibitor, LY2510924, in combination with FLT3 inhibitors in preclinical models of AML with FLT3-ITD mutations (FLT3-ITD-AML). Quizartinib, a potent FLT3 inhibitor, induced apoptosis in FLT3-ITD-AML, while LY2510924 blocked surface CXCR4 without inducing apoptosis. LY2510924 significantly reversed stroma-mediated resistance against quizartinib mainly through the MAPK pathway. In mice with established FLT3-ITD-AML, LY2510924 induced durable mobilization and differentiation of leukemia cells, resulting in enhanced anti-leukemia effects when combined with quizartinib, whereas transient effects were seen on non-leukemic blood cells in immune-competent mice. Sequencing of the transcriptome of the leukemic cells surviving in vivo treatment with quizartinib and LY2510924 revealed that genes related to TGF-β signaling may confer resistance against the drug combination. In co-culture experiments of FLT3-ITD-AML and stromal cells, both silencing of TGF-β in stromal cells or TGF-β-receptor kinase inhibitor enhanced apoptosis by combined treatment. Disruption of the CXCL12/CXCR4 axis in FLT3-ITD-AML by LY2510924 and its negligible effects on normal immunocytes could safely enhance the potency of quizartinib, which may be further improved by blockade of TGF-β signaling.

## 1. Introduction

Mutations in the FMS-like tyrosine kinase 3 gene (FLT3), involved in regulating survival, proliferation, and differentiation of hematopoietic stem/progenitor cells [1], are common in acute myeloid leukemia (AML). These alterations confer inferior response to chemotherapy and poor overall survival, particularly when they occur as internal tandem duplications (ITD) in the juxtamembrane domain whereas activating point mutations in the second tyrosine kinase domain have no significant impact on outcomes [2]. FLT3-ITD mutations constitutively activate phosphatidyl-inositol 3-kinase (PI3K)/AKT, mitogen-activated protein kinase (MAPK)/extracellular signal-regulated kinase (ERK), and signal transducer and activator of transcription 5 (STAT5) pathways and result in uncontrolled cell proliferation and cell survival [3,4]. FLT3-ITD mutations also suppress myeloid transcription factors PU.1 and CCAAT/enhancer-binding protein α, which blocks myeloid differentiation [5,6]. A number of inhibitors of FLT3 signaling have been identified and are being tested in clinical trials, both alone and in combination with chemotherapy [7]. Currently, three FLT3 inhibitors, including midostaurin, gilteritinib, and quizartinib have been approved for the treatment of FLT3-ITD positive AML (FLT3-ITD-AML) in each indication. However, while inhibitor monotherapy produces clinical responses, the responses are usually incomplete and transient, and resistance develops rapidly [7].

While genomic and epigenetic mechanisms of drug resistance are rapidly being uncovered and targeted, leukemic cells are also critically dependent upon the microenvironment in the bone marrow (BM) or stem cell niche. It has been well established that BM stromal cells convey drug resistance in AML [8,9,10]. Targeting constitutively activated FLT3 with tyrosine kinase inhibitors in AML eradicates blasts in the periphery but not in the BM, suggesting a protective effect of the marrow niche [11]. Stromal niche cells protect early leukemic FLT3-ITD progenitor cells against FLT3 inhibitors [12]. C-X-C chemokine receptor type 4 (CXCR4) and its ligand, C-X-C motif chemokine 12 (CXCL12), are key mediators of leukemia/stroma interaction. CXCL12 is produced in the BM microenvironment, activates CXCR4 on leukemic cells, facilitates leukemia cell trafficking and homing in the BM microenvironment, and maintains close contact of leukemic cells with stromal cells and the extracellular matrix that constitutively generate growth-promoting and anti-apoptotic signals [13]. Indeed, high CXCR4 expression on AML blasts is known to be associated with poor prognosis [14,15]. Moreover, FLT3-ITD-AML has demonstrated enhanced CXCL12/CXCR4 signaling through upregulation of the cell surface expression of CXCR4 [14,16], which suggests that the CXCL12/CXCR4 axis may influence responsiveness to therapy and confer the poor prognosis of FLT3-ITD-AML [16]. The correlation of CXCR4 with FLT3 was also demonstrated at transcriptomic profiles of AML in The Cancer Genome Atlas (Figure S1). Several groups have tested CXCR4 inhibitors, including small molecule inhibitors (plerixafor and AMD3465), synthetic peptides (LY2510924, BL-8040, and E5), monoclonal antibodies (ulocuplumab and PF-06747143), and lipopolymer complexes of siRNA, all of which were shown to enhance the anti-leukemic effects of chemotherapy in preclinical models [11]. However, there have been concerns regarding the increased toxicity to normal hematopoiesis based on the widespread expression of CXCR4 on hematopoietic cells [17].

Our group showed that the potent peptidic CXCR4 inhibitor, LY2510924, had anti-leukemia efficacy as a single agent and was strongly synergistic with chemotherapy in vivo [10]. The recent initial report of a phase I study found that the combination of LY2510924, idarubicine, and cytarabine, was safe in relapsed AML patients [18]. Given the proven importance of the CXCL12/CXCR4 axis in AML, particularly with FLT3-ITD, and the rapid emergence of resistance to FLT3 inhibitors, FLT3-ITD-AML might represent the subset of AML that derives the most benefit from the combination of FLT3 and CXCR4 inhibitors. In this study, we investigated the efficacy and molecular mechanisms for the strategy to overcome the extrinsic resistance to FLT3 inhibitors in FLT3-ITD-AML by disrupting the CXCL12/CXCR4 axis with a novel CXCR4 inhibitor, LY2510924.

## 2. Results

### 2.1. FLT3 Inhibitors Induce Apoptosis in FLT3-ITD-AML While LY2510924 Blocks Surface CXCR4 and Inhibits Proliferation Rather Than Causing Apoptosis

**Figure 1:**
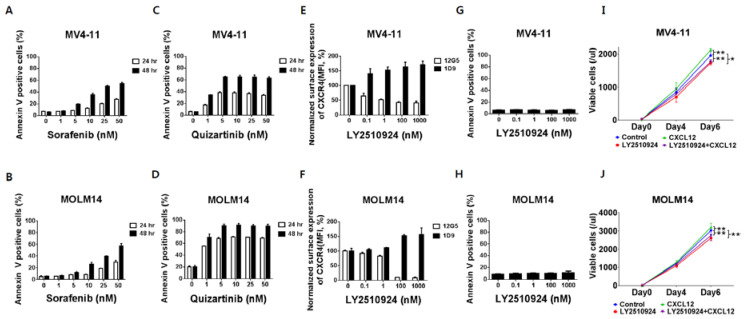
FLT3 inhibitors induce apoptosis in leukemic cells with FLT3-ITD while LY2510924 blocks surface CXCR4 and inhibits proliferation rather than causing apoptosis. (**A**,**B**) Both MV4–11 (**A**,**C**,**E**,**G**) and MOLM-14 (**B**,**D**,**F**,**H**) cells were cultured with different concentrations of sorafenib (**A**,**B**) and quizartinib (**C**,**D**) for 24 and 48 h, or LY2510924 (**E**,**F**) for 150 min. Both MV4–11 and MOLM-14 cells (3 × 10^4^/mL) were grown in 2% fetal bovine serum containing RPMI in the presence or absence of 100 ng/mL CXCL12, with or without daily treatment with 1 µM LY2510924 for up to seven days. Flow cytometry using annexin V^+^/DAPI^+^ staining and counting beads was used to assess the percentage or number of apoptotic cells (**A**–**D**,**G**–**J**). Surface CXCR4 was measured by flow cytometry with antibody 12G5 (**E**,**F**), which was blocked by receptor occupancy with LY2510924. The results are expressed as the percentage change in mean fluorescent intensity (MFI) compared to control (untreated) cells. All results are expressed as the mean ± SD. * *p* < 0.05, ** *p* < 0.01.

To characterize the effects of CXCR4 blockade by LY2510294 with FLT3 inhibitors, we used FLT3-ITD-AML cell lines MV4–11 and MOLM-14, which express high levels of CXCR4 on their cell surface. The FLT3 inhibitors, sorafenib and quizartinib, induced apoptosis with higher potency of quizartinib than sorafenib, consistent with its superior activity and specificity (Figure 1A–D) [19]. Flow cytometry results showed that LY2510924 partially reduced binding of the anti-CXCR4 antibody 12G5 to surface CXCR4 in a concentration-dependent fashion in FLT3-ITD-AML MV4–11 and completely in MOLM-14 cells (Figure 1E,F). LY2510924 induced moderate upregulation of cell surface CXCR4 as measured by the 1D9 antibody, which binds to a different CXCR4 epitope (Figure 1E,F), consistent with published data that the accumulation of CXCR4 on the cell surface is caused by inhibition of CXCL12-induced CXCR4 internalization [20]. In contrast to the FLT3 inhibitors, LY2510924 did not induce apoptosis in vitro (Figure 1G,H) but moderately inhibited proliferation of the FLT3-ITD-AML cells (Figure 1I,J).

### 2.2. CXCR4 Inhibition by LY2510924 Significantly Reverses Stroma-Mediated Resistance to Quizartinib In Vitro, Mainly Through the MAPK Pathway

**Figure 2:**
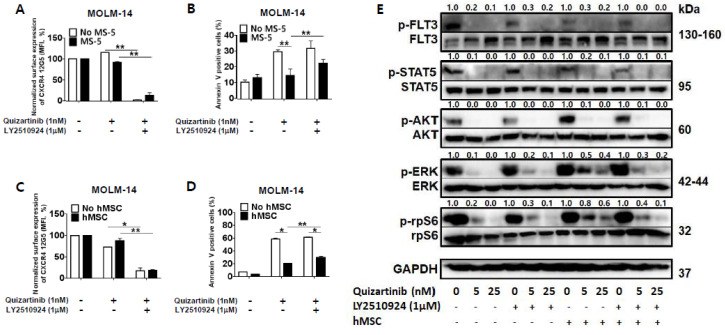
LY2510924 reverses stroma-mediated resistance to quizartinib mainly through the MAPK pathway. (**A**–**D**) MOLM-14 cells were cultured alone (mono-culture) or co-cultured with stromal cells (MS-5 and hMSC from FLT3-ITD-AML) as indicated in the Materials and Methods. Mono-cultured and co-cultured cells were treated for 72 h with 1.0 nM quizartinib in the presence or absence of 1 µM LY2510924. Surface CXCR4 12G5 staining (**A**,**C**) and percentages of apoptotic cells (**B**,**D**) were assessed by flow cytometry. All results are expressed as the mean ± SD. * *p* < 0.05, ** *p* < 0.01. (**E**) After four hours of incubation with different doses of quizartinib in the presence or absence of 1 µM LY2510924, MOLM-14 cells were harvested, and phosphorylation of FLT3, STAT5, AKT, ERK, and rpS6 (ribosomal protein S6) were detected by Western blot analysis. GAPDH was used as a loading control.

To determine the combined effects and mechanisms of the CXCR4 blockade by LY2510294 with FLT3 inhibitors, we next tested whether CXCR4 inhibition by LY2510924 could overcome stroma-mediated protection against quizartinib in FLT3-ITD mutated AML cells in vitro by co-culturing MOLM-14 cells with MS-5 stromal cells or hMSC from FLT3-ITD-AML (Table S1) for three days. CXCR4 binding to 12G5 antibody was blocked by LY2510924 (Figure 2A,C) in both culture systems, with or without stromal cells. The quizartinib-induced apoptosis of AML cells was significantly reduced by stromal cells, and this protective effect of stromal cells was reduced by LY2510924 (Figure 2B,D).

Given that LY2510924 reduced stroma-mediated resistance to quizartinib, we next tested the effects of LY2510924/FLT3 inhibitor combination on FLT3 signaling by studying the phosphorylation of FLT3 and downstream proteins in the FLT3 signaling pathway. FLT3 inhibition by quizartinib induced de-phosphorylation of FLT3 and downstream proteins in mono-culture system (Figure 2E). Co-culture with stromal cells had no significant effect on the phosphorylation of FLT3 by quizartinib. In terms of the downstream proteins, different effects of co-culture with stromal cells on the expression of AKT and ERK were seen in response to FLT3 inhibition. In the presence of stromal cells, FLT3 inhibition still induced AKT de-phosphorylation, but ERK phosphorylation was not fully inhibited, consistent with previous findings by Yang et al. [21]. However, CXCR4 inhibition by LY2501924 induced de-phosphorylation of ERK, even in the presence of stromal cells, which was supported by inhibition of phosphorylation of the ribosomal protein S6 (rpS6). rpS6 is known to be directly phosphorylated through activation of p90 ribosomal S6 kinase by MAPK pathway in FLT3-ITD-AML [22] as well as activation of p70-S6 kinase 1 by PI3K/AKT/mTOR pathway. Thus, the anti-apoptotic effects of BM stroma appear to correlate with the persistent activation of ERK, which could be effectively reversed by disruption of the CXCL12/CXCR4 axis by LY2510924.

### 2.3. LY2510924 Enhances Anti-Leukemia Effects in Combination with Quizartinib In Vivo

**Figure 3:**
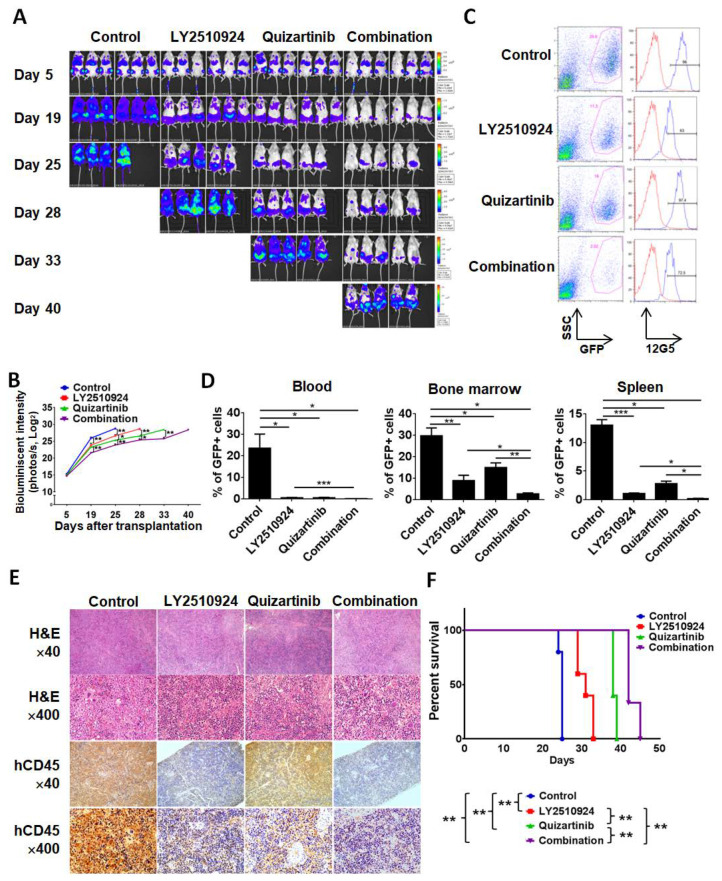
LY2510924 enhances anti-leukemia effects in combination with quizartinib in vivo. MOLM-14/Luc/GFP cells (1.0 × 10^6^ cells per mouse) were intravenously injected into NSG mice. (**A**–**F**) After confirming leukemia engraftment by bioluminescence imaging, the mice were divided into four groups (10 mice per group) and treatment began on day 5 until day 25: Control (no treatment), quizartinib (2.5 mg/kg, oral, daily), LY2510924 (2.5 mg/kg, subcutaneous, daily), or the combination of quizartinib with LY2510924. Quizartinib was administered three hours after LY2510924 administration. Representative mice from each group were subjected to serial bioluminescence images (**A**) and intensity quantitation (**B**) on days 5, 19, 25, 28, 33, and 40 after leukemic cell injection. (**C**–**E**) Three representative mice per group were euthanized on day 20 to compare leukemic burdens in each group. Cells from the BM, spleen, and blood were analyzed by flow cytometry and the expression of CXCR4 12G5 in BM from a representative mouse (**C**) shows CXCR4 blockade in GFP-positive cells in LY2510924-treated groups (single or combination). The proportion of GFP-positive cells by flow cytometry (**D**) and human CD45-positive cells by immunohistochemical analysis (**E**) to identify leukemic cells were compared between each group. The overall survival rate in each group (7 mice per group) was estimated by the Kaplan–Meier method (**F**). The results are expressed as the mean ± SEM. * *p* < 0.05, ** *p* < 0.01, *** *p* < 0.001.

To test the anti-leukemia efficacy of LY2510924 in combination with quizartinib in vivo, we injected MOLM-14 cells into non-irradiated NSG mice. Mice were randomized into four cohorts, which received the following treatment on day 5 post cell injection: Vehicle, quizartinib only, LY2510924 only, or the combination of quizartinib and LY2510924 for 21 days. Bioluminescence imaging (BLI) demonstrated significantly reduced leukemic burden in all treated groups compared to controls (Figure 3A,B). Single agent therapy with quizartinib and LY2510924 reduced AML tumor burden, showing comparable effects on day 19, then quizartinib became more effective than LY2510924, and the combination was most effective. On day 20, after two weeks of daily treatment, three mice were euthanized in each group, and flow cytometry of circulating leukemic cells, in BM, and spleens revealed significant blockade of CXCR4 12G5 staining by LY2510924 in vivo (Figure 3C and Figure S2). Flow cytometry data on day 20 further demonstrated less leukemic infiltration in all treated groups, the lowest infiltration in the combination group, and no significant difference in infiltration between quizartinib- and LY2510924-treated mice (Figure 3D), consistent with BLI data on day 19 (Figure 3A,B). These findings were also demonstrated in histology and immunohistochemical staining of tissues for human CD45 (Figure 3E). Survival was prolonged in all treated mice compared to the controls and combination therapy was the most effective in extending the survival of the treated animals (Figure 3F).

### 2.4. LY2510924 Induces Durable Mobilization of Leukemia Cells and Differentiation Without Causing Apoptosis In Vivo, with Only Transient Effects on Non-Leukemic Blood Cells

**Figure 4:**
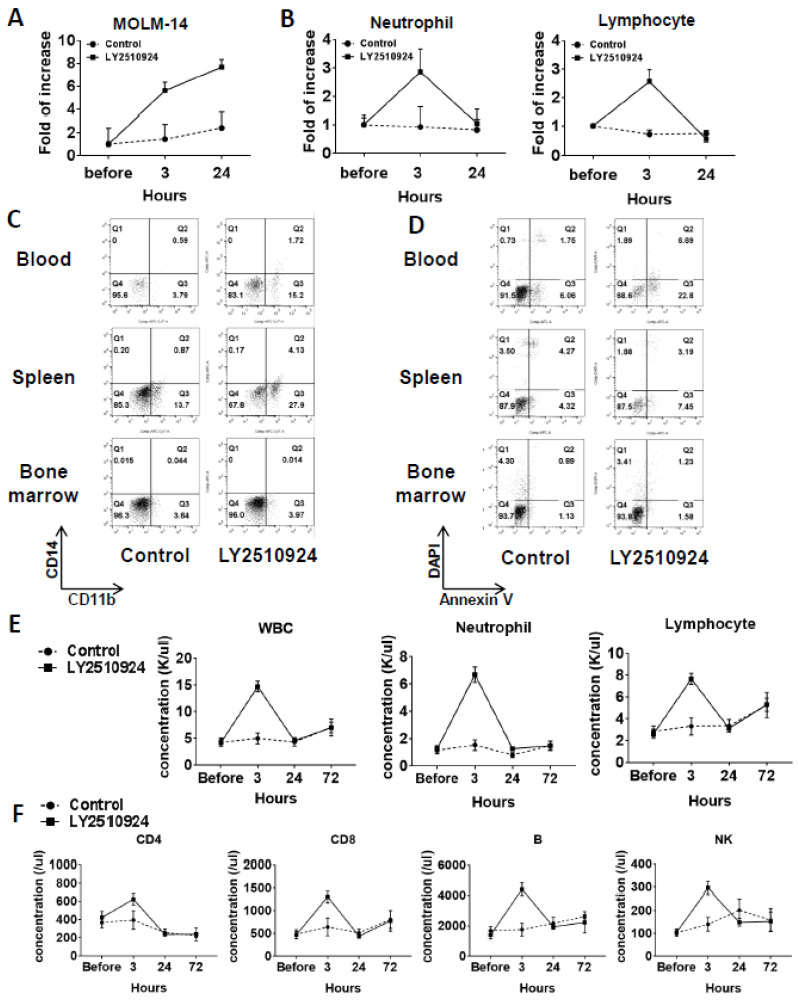
LY2510924 induces durable mobilization of leukemia cells and differentiation without causing apoptosis in vivo, whereas it causes transient effects on non-leukemic blood cells. (**A**–**D**) In a separate group from mice xenografted with MOLM-14/Luc/GFP cells (1.0 × 10^6^ cells per mouse), the percentages of circulating MOLM-14/Luc/GFP cells analyzed by flow cytometry (**A**) and the neutrophils and lymphocytes by the HemaVet 950 Auto Blood Analyzer (**B**) before and three or 24 h after the first LY2510924 (2.5 mg/kg, subcutaneous, daily) injection on day 25 in three mice were compared to those in untreated mice (*n* = 3). After daily treatment of LY2510924 for two days, CD11b and CD14 expression (**C**) and the degree of apoptosis in GFP-positive cells (**D**) in the BM, spleen, and blood were compared to the untreated group. To further explore the different effects of normal blood cells in immune-competent mice, C57/BL/6 mice were treated with LY2510924 (2.5 mg/kg, subcutaneous, daily) and blood cell counts were analyzed (**E**) and flow cytometry was conducted (**F**) on each immune cell subsets.

In a separate group of MOLM-14/Luc/GFP xenografted mice, on day 25 after cell injection, we observed the mobilization of leukemic cells at 3 and 24 h after LY2510924 injection caused by the sustained blockade of CXCR4 (Figure 4A and Figure S2). Circulating leukemia cells were significantly increased by LY2510924 at three hours after LY2510924 administration compared to controls and further increased 24 h after treatment (Figure 4A). In contrast, neutrophil and lymphocyte counts were increased at three hours after treatment but returned to near baseline levels by 24 h (Figure 4B). The CD11b+ expression of MOLM-14 cells was increased in blood (2.7 fold) and spleen (4 fold) after daily treatment of LY2510924 for two days (Figure 4C), suggesting myeloid differentiation of leukemic cells by the CXCR4 blockade, while no evidence of apoptosis induction in vivo was observed (Figure 4D), consistent with the in vitro data (Figure 1H). Treatment with LY2510924 for five days did also not induce apoptosis in circulating MOLM-14 cells (Figure S3). To further explore the effects on normal blood cells in immune-competent mice, we treated C57/BL/6 mice with LY2510924. Analysis of blood cell counts showed transient mobilization on white blood cells, including neutrophils and lymphocytes (Figure 4E), consistent with those of the MOLM-14/Luc/GFP xenograft model (Figure 4B). In addition, flow cytometry for specific immune subsets of lymphocytes, including CD4, CD8, B, and NK cells, showed the same patterns of transient mobilizations (Figure 4F). 

### 2.5. Transcriptome Analysis of the Differentially Expressed Genes upon Combination of FLT3 and CXCR4 Inhibitors in AML

**Figure 5:**
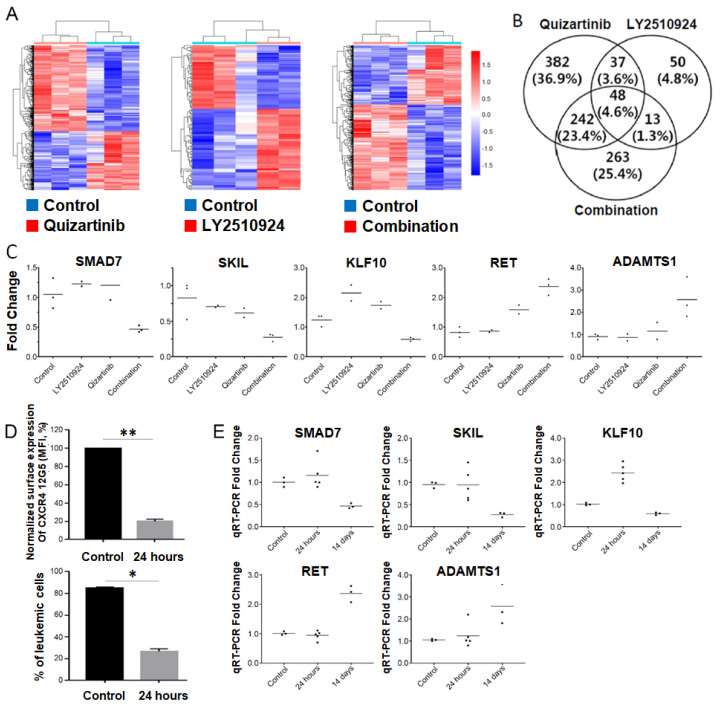
Suppression of SMAD7, SKIL, and KLF10, and upregulation of RET and ADAMTS1 were found in the combined treatment group. (**A**–**C**) To elucidate the molecular effects of the combination of FMS-like tyrosine kinase 3 gene (FLT3) and C-X-C chemokine receptor type 4 (CXCR4) inhibitors in vivo, the global gene expression profiles with leukemia cells from the BM by FACS using GFP of the MOLM-14/Luc/GFP xenograft model (three controls, three quizartinib only, two LY2510924 only, and three combined treatment) euthanized on day 20 in the experiments shown in Figure 3 were examined using whole transcriptome sequencing. (**A**) Heatmap of the differentially expressed genes detected in the quizartinib-treated group (left), the LY2510924-treated group (middle), or the combination group (right) compared with controls. Red and blue represent the up-regulated and down-regulated genes, respectively. (**B**) Venn diagram of differentially-expressed genes (DEGs) detected in the three different groups. (**C**) Expression levels of five genes measured by RT-qPCR. The relative gene expression levels of each gene were normalized to the GAPDH housekeeping gene. *Y*-axis represents relative gene expression levels (fold change) based on the median of controls as the calibrator. (**D**,**E**) To examine the longitudinal changes in five candidate genes after combined treatment with FLT3 and CXCR4 inhibitors, separate experiments with the MOLM-14/Luc/GFP xenograft model were performed. The mice began daily treatment with quizartinib and LY2510924 on day 17, after sufficient involvement of leukemic cells in the blood. (**D**) Five mice were euthanized at 24 h after treatment as a group for early change and three untreated mice were used as controls. Leukemic cells were isolated from the BM by FACS using GFP. BM sample at 24 h after combined treatment showed successful CXCR4 blockade by LY2510924 in vivo and reduced numbers of leukemic cells. (**E**) The expression levels of the five genes were shown over time with the combined treatment. The expression levels two weeks after treatment were taken from data of transcriptome sequencing validation (Figure 5C). The results are expressed as the mean ± SEM. * *p* < 0.05, ** *p* < 0.01.

To elucidate the molecular effects of the combination of FLT3 and CXCR4 inhibitors in vivo, we examined the global gene expression profiles of tumors from BM in four groups of MOLM-14/Luc/GFP xenografted mice (controls (*n* = 3), quizartinib (*n* = 3), LY2510924 (*n* = 2), and combination (*n* = 3)) using whole transcriptome sequencing. When each treated group was compared with untreated controls, a total of 709, 148, and 566 differentially-expressed genes (DEGs) was identified in the quizartinib, LY2510924, and combination-treated groups, respectively (Tables S2 and S3). Unsupervised hierarchical clustering analysis of the DEGs showed clearly distinct patterns between the treated and untreated groups (Figure 5A). In addition, 263 genes were uniquely detected in the combination group, suggesting that they may be associated with resistance or synergistic effects in the surviving cells after treatment (Figure 5B). To gain insights into the biological implications of the uniquely detected DEGs in the combination group, we performed gene ontology and pathway-level analysis using the 263 DEGs (141 upregulated genes and 122 downregulated genes). The uniquely detected DEGs in the combination group were significantly associated with regulation of cell migration (downregulated DEGs, *p* = 1.4 × 10^−6^, and upregulated DEGs, *p* = 0.005); negative regulation of transforming growth factor beta (TGF-β) receptor signaling pathway (downregulated DEGs, *p* = 0.043); and negative regulation of cell proliferation (downregulated DEGs, *p* = 0.053) (Table S4).

Among the 263 DEGs uniquely detected in the combination group, we selected five genes, three down-regulated (SMAD7, SKIL, and KLF10) and two up-regulated genes (RET and ADAMTS1), which met criteria for both top ten genes based on statistical significance and genes reported to be associated with cancer. Modulation of these genes were successfully validated by qRT-PCR (Figure 5C). All five candidate genes showed a strong positive correlation between whole transcriptome sequencing and qRT-PCR, indicating the robustness of the whole transcriptome sequencing analysis. Next, to clarify whether these candidate genes played a role in synergism or resistance, we examined the early changes in each gene after the combined treatment of FLT3 and CXCR4 inhibitors. Using the MOLM-14/Luc/GFP xenograft model, mice were administered daily treatment with FLT3 and CXCR4 inhibitors after achieving high levels of leukemia chimerism in the blood (Figure S4A). Five mice were euthanized at 24 h after treatment as an early change group and three untreated mice were used as controls. Leukemic cells were isolated from the BM by fluorescence-activated cell sorting using GFP. BM samples showed successful CXCR4 blockade of quizartinib and LY2510924 in vivo (Figure 5D). qRT-PCR validation at the different time points revealed that all candidate genes, except KLF10, which was up-regulated at early but down-regulated at later time point, were not significantly up- or down-regulated at the early time point (24 h), suggesting that the biologic effects of these genes at later time point could be linked to the resistance and persistence of AML cells after combination therapy (Figure 5E).

### 2.6. Silencing of TGF-β Signaling Reduces the Stroma-Mediated Protective Effects and Further Enhances Apoptosis by the Combined Treatment

**Figure 6:**
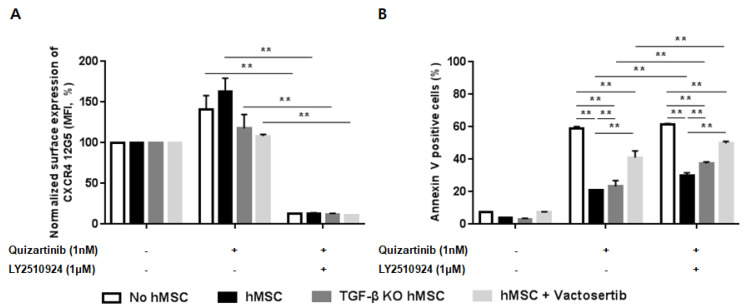
Silencing of transforming growth factor beta (TGF-β) signaling reduces the stroma-mediated protective effects and further enhances apoptosis by the combined treatment. (**A**,**B**) MOLM-14 cells were cultured alone (mono-culture) or co-cultured with human mesenchymal stromal cells (hMSC) and TGF-β knock-down hMSC (TGF-β KD hMSC) as indicated in the Materials and Methods. Mono-cultured and co-cultured cells were treated for 72 h with 1.0 nM quizartinib in the presence or absence of 1 µM LY2510924. A group with mono-cultured cells and co-cultured cells with hMSC were also treated with 1 µM vactosertib. Surface CXCR4 12G5 staining (**A**) and percentages of apoptotic cells (**B**) were assessed by flow cytometry. All results are expressed as the mean ± SD. ** *p* < 0.01.

To investigate the role of TGF-β signaling as a resistance mechanism to the combination of FLT3 and CXCR4 inhibitors informed by the transcriptome analysis, we examined the effects of the combined treatment in hMSC in the presence/absence of TGF-β signaling. Co-culture of MOLM-14 cells with hMSC and TGF-β knock-down hMSC (TGF-β KD hMSC) showed effective blockade of CXCR4 by LY2510924 (Figure 6A) in both culture systems. Figure 6B demonstrated significantly enhanced apoptosis by quizartinib in AML cells co-cultured with TGF-β KD hMSC compared to wild-type hMSC, suggesting reduced stroma-mediated protective effects by silencing TGF-β signaling. Moreover, the combined quizartinib//LY2510924 treatment further enhanced apoptosis in TGF-β KD hMSC compared to wild type hMSC co-cultures (Figure 6B). We also observed the same results with vactosertib (a TGF-β receptor I kinase inhibitor) in co-culture of MOLM-14 cells with hMSC, in which TGF-β blockade by vactosertib enhanced apoptosis by quizartinib and even more by combined treatment (Figure 6A,B).

## 3. Discussion

The current study demonstrated that a novel peptidic CXCR4 inhibitor, LY2510924, effectively disrupted the CXCL12/CXCR4 axis and reversed microenvironment-mediated resistance to the FLT3 inhibitor, quizartinib, mainly through downregulation of persistent MAPK activation, which enhanced the sensitivity of FLT3-ITD-AML cells to FLT3 inhibitors in vitro and in vivo. These observations indicate that targeting the CXCL12/CXCR4 axis is a promising strategy to overcome extrinsic resistance by the protective BM microenvironment in FLT3-ITD-AML through sensitizing leukemic cells to FLT3 inhibitors, contrasting diverse combination strategies focusing on intrinsic resistance mechanisms, such as the acquisition of point mutations in ATP binding regions of the FLT3 kinase domain, mutations in other kinases, the upregulation of parallel pro-survival pathways, upregulation of the FLT3 receptor, and activation of antiapoptotic proteins [23]. The inability to induce apoptosis by LY2510924 alone clearly indicates the sensitizing efficacy of it by disrupting the CXCL12/CXCR4 axis, in contrast with other classes of CXCR4 inhibitors able to induce apoptosis, such as BL-8040[24] and monoclonal antibodies targeting CXCR4 [25,26].

Previous reports have presented a novel strategy of sensitizing AML cells to cytotoxic chemotherapy using different classes of CXCR4 inhibitors, including AMD3465 [9], LY2510924 [10], BL-8040 [24], ulocuplumab [25], and PF-06747143 [26], some of which are in early phase clinical trials [11]. Given the cross-talk between CXCR4 expression and FLT3-ITD, supported by the activation of CXCR4 signaling by FLT3-ITD and higher CXCR4 expression in FLT3-ITD-AML [14,16], our group first demonstrated the proof of concept that a small molecular CXCR4 inhibitor, AMD3465, could enhance anti-leukemia effects of a multi-kinase inhibitor, sorafenib, in a Ba/F3-ITD murine tumor model [9]. This was borne out in a Phase I clinical trial of G-CSF, plerixafor and sorafenib in FLT3-mutated relapsed/refractory AML (NCT00943943), where high response rates were seen even in patients who failed FLT3 inhibitors before [27]. The current study revealed the synergistic effects and mechanisms of combinatorial activity of a novel peptidic CXCR4 inhibitor, LY2510924, with a potent selective FLT3 inhibitor, quizartinib. PI3K/AKT and MAPK/ERK pathways are key signaling pathways that promote leukemic cell survival [28,29] and are known to be linked to both the CXCL12/CXCR4 axis and FLT3 pathways [30,31]. Despite shared AKT and ERK signaling in FLT3 and CXCR4 downstream pathways, BM stroma-mediated resistance to quizartinib in FLT3-ITD-AML was mediated through persistent activation of ERK, not AKT, consistent with a previous report under different co-culture conditions [21]. The persistent activation of MAPK pathway as a resistance mechanism to FLT3 inhibitor-induced apoptosis was previously demonstrated by the acquisition of activating RAS mutations and the persistent activation of MAPK/ERK in MOLM-14 cells resistant to various FLT3 inhibitors [32], and the clonal selection of RAS mutations after FLT3 inhibition by gilteretinib in a clinical trial [33]. Our data suggest that BM blasts additionally maintain ERK activity via CXCR4 signaling, despite suppression of the FLT3 pathway (bypassing the FLT3 receptor), which may manifest clinically as incomplete clearance of BM blasts by FLT3 inhibitors while the circulating blasts are eliminated [11]. CXCR4 blockade by LY2510924 inhibited the phosphorylation of ERK, which partially abrogated the stroma-mediated bypass of the FLT3 receptor, enhanced the apoptosis by quizartinib in co-culture conditions and facilitated anti-leukemia effects in vivo. Since the decreased pro-survival signaling exclusively through the ERK pathway is a mechanism of sensitization of FLT3-ITD-AML to FLT3 inhibitors through CXCR4 blockade, the combination of potent FLT3 inhibition and MEK inhibition could represent a promising strategy for the treatment of FLT3-ITD-AML, which was supported by previous data demonstrating anti-leukemia activity of a MEK1 and FLT3 dual inhibitor, E6201, in AML cells resistant to FLT3 inhibition [34].

With respect to mechanisms of action of LY2510924, physical mobilization of FLT3-ITD-AML blasts from their protective BM microenvironment and induction of differentiation were demonstrated in this study, in agreement with data from the non-FLT3-ITD-AML models in our previous study [10]. These observations are supported by reports of other CXCR4 inhibitors, such as plerixafor [35], BL-8040 [24], and anti-CXCR4 antibody [25,26], in various subtypes of AML. Anti-CXCR4 antibody-induced apoptosis was also demonstrated as one of the mechanisms of anti-leukemia effects [25,26]. Abraham et al. recently reported an additional apoptosis-inducing mode of action by another peptidic CXCR4 inhibitor, BL-8040, in an FLT3-ITD mutated MV4–11 xenograft model [24]. In contrast, in this study, LY2510924 did not induce apoptosis but moderately inhibited the growth of FLT3-ITD-AML cells. The direct cytotoxic effects of BL-8040 may not be the main mechanism of the anti-leukemia effects because they were evident only at higher concentrations (10–20 μM) in vitro [24]. Moreover, cytotoxic effects on CXCR4-expresssing immunocytes inducing unwanted immunosuppression could be a potential cause of toxicity in immunocompetent subjects. These effects should be further investigated in immunocompetent models or clinical trials. In this respect, our data clearly demonstrated the transient effects of LY2510924 on non-leukemic blood cells in NSG mice and immunocompetent mice, contrasting with durable effects on leukemic cells in MOLM-14 xenograft models, suggesting a potential safety and therapeutic window in future clinical trials. Further investigations for short- or long-term effects of LY2510924 on hematopoietic stem cells in animal models or clinical trials will give an answer to the safety issue.

In addition to the aforementioned modes of action for the CXCR4 blockade, we identified potential resistance mechanisms for the combination of FLT3 and CXCR4 inhibitors through the whole transcriptome sequencing analysis of xenograft models. The expression levels of SMAD7, SKIL, and KLF10 were significantly downregulated by the combined treatment, which was observed only late into treatment, suggesting a role in resistant cells surviving therapy. SMAD7 and SKIL are well known as negative regulators of TGF-β signaling [36,37], and KLF10 is believed to play a crucial role as a tumor suppressor with unique tissue-specific functions mediated by TGF-β signaling [38]. These observations indicate the possibility of combined treatment-induced activation of TGF-β signaling, also supported by gene ontology and pathway-level analysis. TGF-β is produced by BM stromal cells and regulates cell proliferation, survival, and apoptosis depending on the cellular context [39]. Moreover, TGF-β1 was reported to induce CXCR4 expression [40,41]. Activation of TGF-β signaling as a resistance mechanism is supported by our previous data that demonstrated a potential role of TGF-β signaling in modulating the sensitivity of AML cells to chemotherapeutic agents, in which the blockade of TGF-β1 enhanced the response to the combination of cytarabine and CXCR4 inhibitor (plerixafor) in vitro and prolonged survival in an in vivo leukemia model [42]. The current study demonstrated that silencing of TGF-β signaling by TGF-β KD hMSC or TGF-β receptor I kinase inhibitor not only reduced the stroma-mediated protective effects against quizartinib but also further enhanced synergistic effects of the combined treatment, suggesting a role for TGF-β blockage in overcoming the resistance against the combination of FLT3 and CXCR4 inhibitors. These findings also indicate the possible causal relationship between MAPK and TGF-β signaling in the acquisition of stroma-mediated resistance, which needs to be further evaluated.

The expression level of RET and ASAMTS1 were also significantly upregulated by the combined treatment. Activating alterations of the RET kinase are therapeutically actionable oncogenic drivers across a variety of cancers [43], but the role of RET in hematologic malignancies is less well defined. Recent studies have demonstrated that RET expression may contribute to leukemogenesis in AML models [44,45]. In a model of FLT3-ITD-AML, activation of RET suppressed autophagy, resulting in stabilization of leukemogenic drivers such as mutant FLT3, important RET effectors [45]. ADAMTS1, one of the extracellular degrading enzymes, plays an essential role in aberrant tissue remodeling of the peritumoral environment but its role in hematologic malignancies is not known. Perturbations in ADAMTS1 evoke significant changes that ultimately promote cancer development and metastatic progression [46]. The potential role of the upregulation of RET and ADAMTS1 as a resistant mechanism needs to be further evaluated.

## 4. Materials and Methods

Please refer to the Supplemental Methods for detailed descriptions.

### 4.1. Cell Lines and Materials

Human FLT3-ITD-AML cell lines, MV4–11 (ATCC, Manassas, VA, USA) and MOLM-14 (DSMZ, Braunschweig, Germany, Table S1), were used in this study. All cell lines were cultured in RPMI 1640 medium supplemented with 10% fetal bovine serum and 1% penicillin-streptomycin. The cells were harvested during the log phase of growth and seeded at a density of 0.2 × 10^6^ cells/mL. LY2510924 was kindly provided by Eli Lilly (Indianapolis, IN, USA), and quizartinib was purchased from ApexBIO (Houston, TX, USA). Sorafenib and vactosertib were purchased from Medchem Express (Monmouth Junction, NJ, USA).

### 4.2. Flow Cytometry and Mouse Blood Cell Count

The expression of various target proteins was analyzed using a LSRFortessa^TM^ flow cytometer (BD Biosciences, San Jose, CA, USA). The harvested cells were stained with antibodies against human and mouse cells targets and an appropriate isotype-matched antibody was used as a negative control. In vivo leukemic cells were isolated using green fluorescent protein (GFP) for AML cell lines. Flow cytometric data were analyzed with FlowJo vX.10 software. The HemaVet 950 Auto Blood Analyzer (Drew Scientific, Inc., Miami Lakes, FL, USA) was used for the blood cell counts in mice.

### 4.3. Western Blot Analysis

Cell lysates were separated on 8% or 10% polyacrylamide gels, transferred to nitrocellulose membranes, incubated with the appropriate antibodies and infrared secondary antibodies (Cell Signaling Technology, Danbers, MA, USA), and quantified by the Chemiluminescence Image Analyzer system (Fusion SL-4 3500; Vilber Lourmat, Collégien, France). The antibodies used were rabbit antihuman FLT3 (8F2), STAT5, phospho-AKT (Ser473), phospho-p44/42 MAPK (Erk1/2)(Thr202/Tyr204), and phospho-S6 ribosomal protein (Ser240/244), and mouse antihuman phospho-FLT3(Tyr591), phospho-STAT5 (Tyr694), p44/42 MAPK(Erk1/2)(L34F12), and S6 ribosomal protein (54D2) (Cell Signaling Technology, Danbers, MA, USA). Glyceraldehyde-3-phosphate dehydrogenase was used as the loading control (Figure S5).

### 4.4. Co-Culture with Stromal Cells

MOLM-14 cells were co-cultured with MS-5 or primary human mesenchymal stromal cells (hMSC). After 72 h of incubation at 37 °C in a humidified atmosphere containing 5% CO_2_, co-cultured cells were harvested and apoptotic MOLM-14 cells were quantified by flow cytometry. For immunoblotting, after four hours of incubation, the MOLM-14 cells were harvested and their lysates were analyzed by immunoblotting for FLT3, STAT5, ERK, S6K, and AKT as described above.

### 4.5. AML Mouse Models

NOD/SCID/IL-2rγnull (NSG, The Jackson Laboratory, Bar Harbor, ME, USA) mice were used for in vivo xenograft experiments. The mice were injected with MOLM-14 cells labeled with Luc/GFP [47] to establish AML. All animal experiments were done in accordance with a protocol approved by the Institutional Animal Care and Use Committee of The Catholic University of Korea (CUMC-2016-0228-05).

### 4.6. RNA Sequencing and Validation of Gene Expression

Whole transcriptome sequencing was performed using the Illumina NovaSeq 6000 platform. Acquisition and processing of the sequencing data were performed as previously described [48]. Gene-level quantification of expression was performed with HTSeq [49], according to the Ensembl transcript annotation (Homo_sapiens_GRCh38.91 version, ftp://ftp.ensembl.org/pub/release-91/gtf/homo_sapiens/). Differentially expressed genes (DEGs) were defined by a false discovery rate of <0.05 and a log2 fold-change of >1 using the edgeR R package [50]. Candidate genes identified with whole transcriptome sequencing were validated by quantitative reverse transcription PCR (qRT-PCR, Table S2).

## 5. Conclusions

Our study showed that LY2510924 effectively disrupted the CXCL12/CXCR4 axis and reversed stroma-mediated extrinsic resistance to FLT3 inhibitors in FLT3-ITD-AML, mainly through the downregulation of stroma-induced MAPK activation. Efficient and durable CXCR4 blockade by LY2510924 induced physical mobilization and differentiation of leukemic cells, translating into enhanced anti-leukemia efficacy. Transient non-deleterious effects on normal hematopoietic cells and lack of apoptosis by LY2510924 may positively contribute to safety in future clinical trials. The contribution of TGF-β signaling and other candidate genes to resistance mechanisms against the combination of FLT3 and CXCR4 inhibitors, once validated, may generate additional strategies to further improve efficacy of this approach.

## Supplementary Materials

The following are available online at https://www.mdpi.com/2072-6694/12/7/1737/s1, Figure S1: Gene correlation analysis, Figure S2: Mobilization of leukemic cells in blood by sustained blockade of CXCR4 by LY2510924 in MOLM-14/Luc/GFP xenograft models, Figure S3: Prolonged treatment with LY2510924 for 5 days did not induce apoptosis in FLT3-ITD-AML cells in peripheral blood of MOLM-14/Luc/GFP xenograft models, Figure S4: Successful blockade of CXCR4 by the combined treatment of quizartinib and LY2510924 in the bone marrow from MOLM-14/Luc/GFP xenograft models, Figure S5: Original whole blots image of Western blot in Figure 2E, Table S1: Characteristics of FLT3-ITD-AML cell lines and human mesenchymal stromal cells from a patient, Table S2: Target-specific primers for qRT-PCR, Table S3: Differentially expressed genes, Table S4: Gene ontology and pathway analyses of uniquely detected DEGs in combination group using DAVID, Supplementary methods.
