# Supplementary material for: CXCR4 Inhibition Enhances Efficacy of FLT3 Inhibitors in FLT3-Mutated AML Augmented by Suppressed TGF-β Signaling
Source: Cancers (Basel). 2020 Jun 30;12(7):1737. doi: 10.3390/cancers12071737 (PMC7407511; doi:10.3390/cancers12071737)
Supplement: Supplementary file 1 [file cancers-12-01737-s001.zip › Supplementary Figures_revised.docx]

Supplementary Materials: CXCR4 Inhibition Enhances Efficacy of FLT3 Inhibitors in FLT3-Mutated AML Augmented by Suppressed TGF-β Signaling

Bo-Reum Kim, Seung-Hyun Jung, A-Reum Han, Gyeongsin Park, Hee-Je Kim, Bin Yuan, Venkata Lokesh Battula, Michael Andreeff, Marina Konopleva, Yeun-Jun Chung and Byung-Sik Cho

**Figure S1. Gene correlation analysis.** There is a positive correlation between expression levels of CXCR4 and FLT3 (P=8.3x10-6). Transcriptomic profiles of AML were obtained from The Cancer Genome Atlas (TCGA) and the Pearson correlation coefficient was calculated on Gene Expression Profiling Interactive Analysis (GEPIA) (1).

1. Z. Tang, C. Li, B. Kang, G. Gao, C. Li, and Z. Zhang. (2017) GEPIA: a web server for cancer and normal gene expression profiling and interactive analyses. Nucleic Acids Research, 45(W1):W98-W102


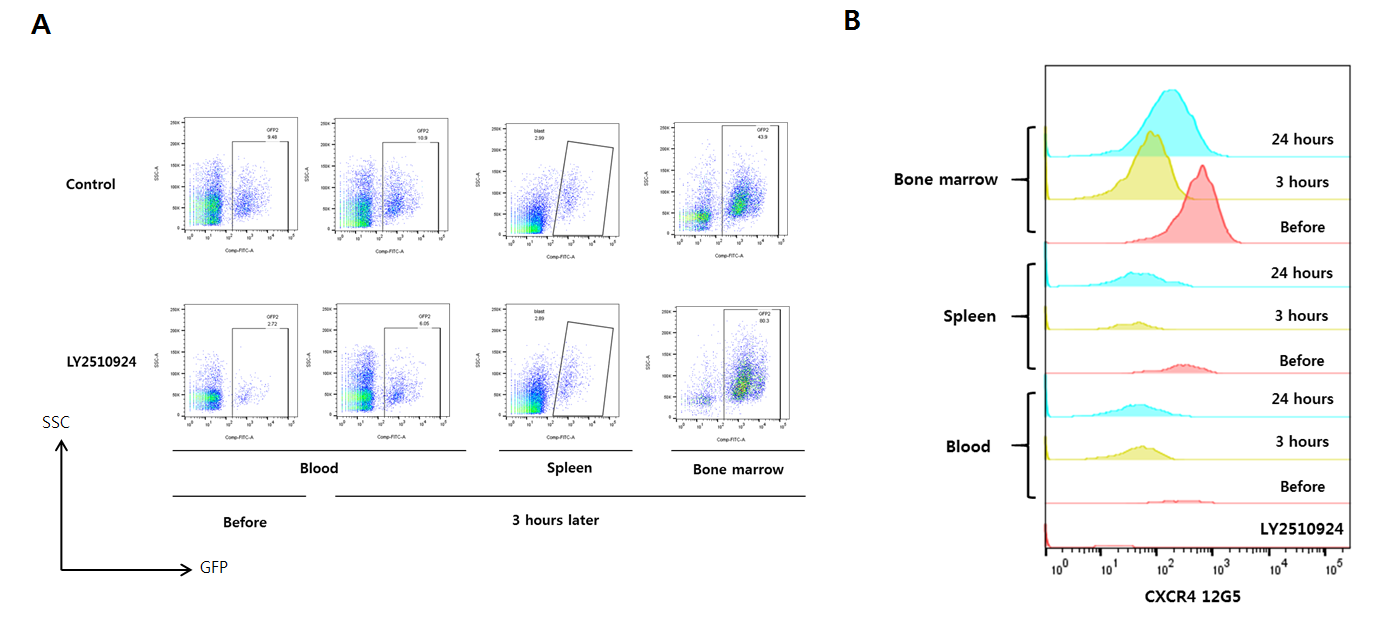


**Figure S2. Mobilization of leukemic cells in blood by sustained blockade of CXCR4 by LY2510924 in MOLM-14/Luc/GFP xenograft models.** In MOLM-14/Luc/GFP xenograft models, on day 25 after cell injection, (A) the mobilization of GFP positive leukemic cells in blood at 3 hours after LY2510924 injection is shown in a representative mouse, which was sacrificed at 3 hours after a single LY2510924 administration. (B) In GFP positive leukemic cells in blood, spleen, and bone marrow, staining for CXCR4 with antibody 12G5 (inversely reflective of receptor occupancy) after a single LY2510924 administration shows effective and sustained blockade of CXCR4 until 24 hours in representative mice.


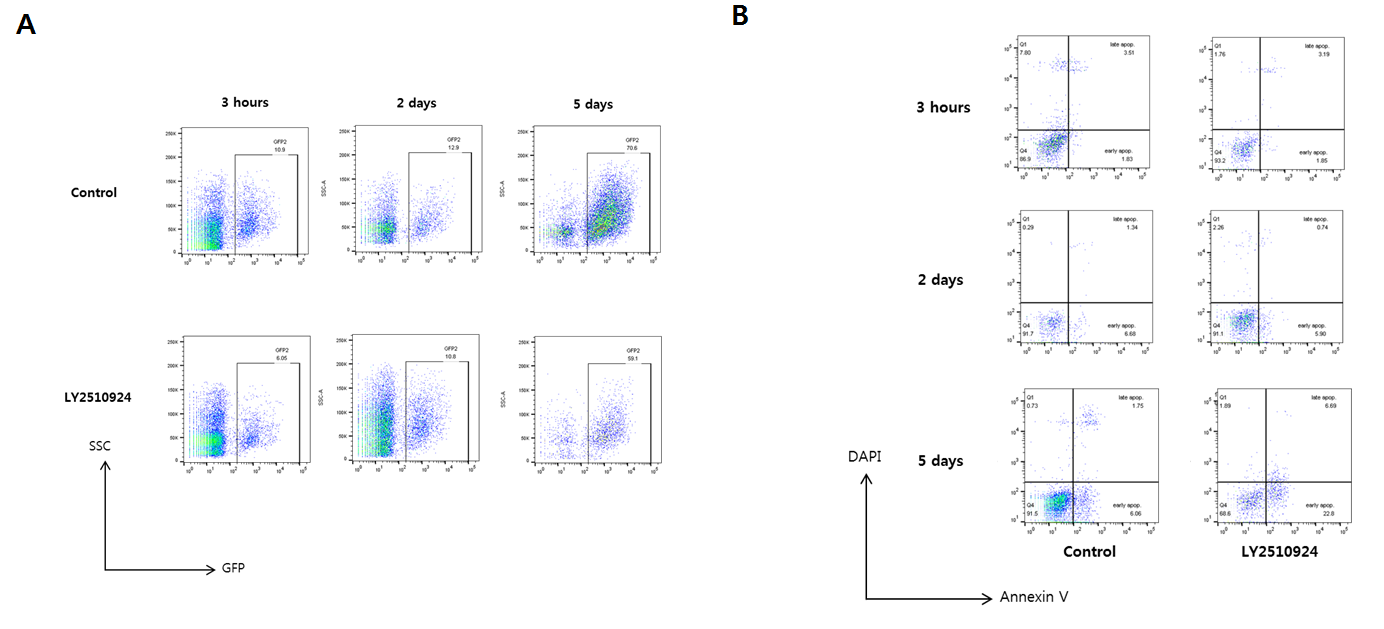


**Figure S3. Prolonged treatment with LY2510924 for 5 days did not induce apoptosis in FLT3-ITD-AML cells in peripheral blood of MOLM-14/Luc/GFP xenograft models.** In MOLM-14/Luc/GFP xenograft models, on day 25 after cell injection, GFP positive leukemic cells in peripheral blood at each time point (A) after daily LY2510924 treatment did not show any difference in Annexin V positive cells by LY2510924 treatment in representative mice (B).


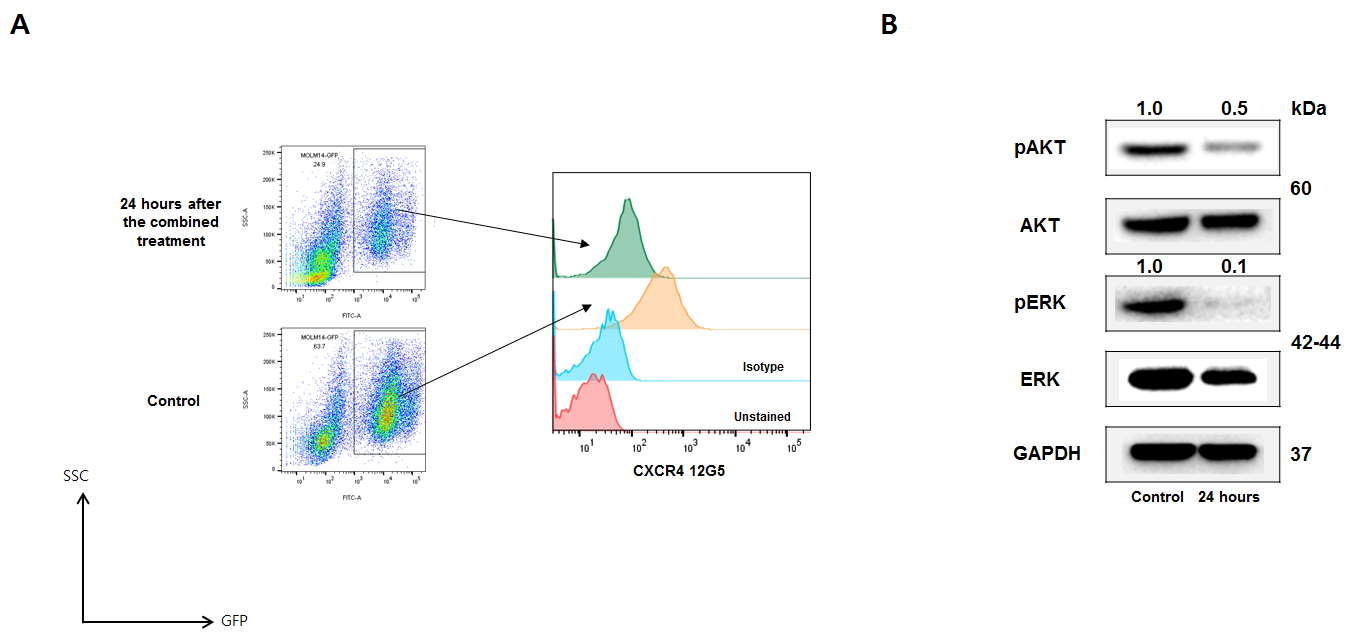


**Figure S4. Successful blockade of CXCR4 by the combined treatment of quizartinib and LY2510924 in the bone marrow from MOLM-14/Luc/GFP xenograft models.** In MOLM-14/Luc/GFP xenograft models, the combined treatment (quizartinib and LY2510924) began on day 17 after cell injection. GFP positive leukemic cells and their CXCR4 12G5 surface expression in bone marrow at 24 hours after the combined treatment compared to an untreated mouse (control) are shown in representative mice.

**Figure S5. Original whole blots image of Western blot in Figure 2E.** (A) Phospho-proteins and GAPDH and (B) total proteins for Figure 2E.
