# Supplementary material for: CXCR4 Inhibition Enhances Efficacy of FLT3 Inhibitors in FLT3-Mutated AML Augmented by Suppressed TGF-β Signaling
Source: Cancers (Basel). 2020 Jun 30;12(7):1737. doi: 10.3390/cancers12071737 (PMC7407511; doi:10.3390/cancers12071737)
Supplement: Supplementary file 1 [file cancers-12-01737-s001.zip › Supplementary Methods_revised.docx]

**Supplemental Methods**

***Antibody lists for flow cytometry***

Harvested cells were stained antibodies against human CXCR4-allophycocyanin (APC; 12G5), CXCR4-phycoerythrin (PE; 1D9), CD45-APC-H7, CD11b-APC-Cy7, CD11c-PE, and CD15-PE-CF594 purchased from BD Biosciences (BD Biosciences, San Jose, CA, USA) and CD14-APC from eBioscience (eBioscience, Waltham, MA, USA). DAPI (Sigma Aldrich, St. Louis, MO, USA) and annexin V-FITC c were used to stain dead and apoptotic cells, respectively. Mouse cells were stained antibodies against mouse CD4-FITC, CD19-PE, NK1.1-BV421, CD184 (CXCR4)-PE purchased from BD Biosciences (BD Biosciences, San Jose, CA, USA), and CD8- PerCP5.5 (eBioscience, Waltham, MA, USA) and CD184 (CXCR4)-Biotin (Miltenyi Biotec, Bergisch Gladbach, Germany).

***Isolation and culture of human mesenchymal stromal cells***

Human mesenchymal stromal cells (hMSC) were isolated from the bone marrow (BM) of normal healthy donors undergoing BM harvest for use in allogeneic transplantation under conformed consent. Each BM specimen was subjected to centrifugation (700 g for 20 minutes at 4°C) over a Ficoll-Hypaque gradient (Sigma-Aldrich, St. Louis, MO, USA) to separate mononuclear cells. After centrifugation, the buffy coat layer was carefully extracted; resuspended in alpha-modified essential medium (Cellgro, Mediatech, Inc., Corning, NY, USA) containing 10% FBS, L-glutamine, 4% pooled human platelet lysate,^1^ and 1% penicillin-streptomycin; and plated at an initial density of 5 × 10^5^ cells/cm^2^. After 3 days, the cultures were washed with PBS (Cellgro, Media Tech Inc., Corning, NY, USA), and the remaining adherent cells were cultured until approximately 80% confluent. The cells were then subcultured at densities of 5,000–6,000 cells/cm^2^. Only third or fourth passage cells were used for the experiments. All BM donors provided written informed consent. Studies involving human materials were conducted according to a protocol approved by the Institutional Review Boards of MD Anderson Cancer Center.

***Production of TGFβ knockdown cells***

TGF-β1 shRNA sequence was used to make TGF-β knockdown hMSC. We ordered the set, total five sequences from Dharmacon, and got the highest knockdown efficacy in #3 (Accessions: NM_000660, XM_011527242; Clone ID: TRCN0000003318; Sequencing Primer: 5' - AAACCCAGGGCTGCCTTGGAAAAG - 3'; Primer runs in the reverse complement direction; Vector Map: pLKO.1). We tested the knockdown efficacy by Q-OCR and ELISA after adding puromycin (1ug/ml) in the culture media for 3-5 days. The MSC was passaged every 3 days. The shRNA control is TRC Lentiviral pLKO.1 Empty Vector Control (Cat# RHS4080). HEK293T cells were used for lenti virus producing. For T25 HEK293T cells (seed the day before transfection), pMD2.G (0.5ug)+psPAX2 (1.5ug)+shRNA plasmid (2ug) were added in 0.2ml Jet Prime buffer, then 8ul Jet Prime Reagent was added, leave room temperature for 10 min. The add to the medium, change medium at the same day, keep culture for 48-72 hours. Collect the supernatant and filter through 0.45um filter, immediately infect the MSC or freeze in -80C. (fresh use is the better way) infect the MSC for 24 hours, then change to regular medium, add puromycin for selection after 24 hours.

***Co-culture with stromal cells for cytotoxicity assay***

MS-5 (DSMZ, Braunschweig, Germany) and hMSC plated at a density of ~2.5 × 10^4^ cells/well in 24-well plates in alpha-modified essential medium (Cellgro, Mediatech, Inc., Corning, NY, USA) supplemented with 20% fetal bovine serum (FBS, Youngin Frontier, Seoul, Republic of Korea) and 1% penicillin-streptomycin (Gibco Laboratories, Grand Island, NY, USA) and MSC growth medium 2 (PromoCell, Heidelberg, Germany), respectively. Twenty-four hours later, the supernatant medium was replaced with 10% FBS–RPMI containing ~1 x 10^5^ leukemia cells pre-incubated for two hours at 37°C either in 10% FBS–RPMI or in the same medium containing 1 µM LY2510924 and/or 1 µM vactosertib ; the cells were then seeded on top of the stromal cells. Twenty-four hours later, quizartinib (ApexBIO, Houston, TX, USA) was added to a 0.5 or 1.0 nM concentration in indicated wells, respectively. After 72 hours of incubation at 37°C in a humidified atmosphere containing 5% CO_2_, cells were harvested with trypsin/ethylenediaminetetraacetic acid (EDTA; Invitrogen, Carlsbad, CA, USA), washed, and resuspended in binding buffer containing annexin V-FITC (BD Biosciences, San Jose, CA, USA). Cells were counterstained with CD45-APC-H7, CXCR4 12G5, and CXCR4 1D9 (BD Biosciences, San Jose, CA, USA), or with respective isotype control antibody, and analyzed by flow cytometry after electronic gating on CD45^+^ cells. The percentage of apoptotic cells was estimated by measuring phosphatidylserine externalization in cells by using annexin V flow cytometry in combination with DAPI (Sigma Aldrich, St. Louis, MO, USA) staining in CD45^+^ cells. The percentage of viable cells was calculated by subtracting annexin V^+^/DAPI^+^ cells from the total CD45^+^ cells. To determine the absolute number of viable cells, 550,000 counting beads were added to each test tube before analysis by a FACS LSR Fortessa machine (BD Biosciences, San Diego, CA, USA)

***Co-culture with stromal cells for immunoblotting***

For the immunoblotting, hMSC plated at a density of 13 × 10^4^ cells/well in 6-well plates in 4 mL [Dulbecco Modified Eagle Medium (DMEM, Gibco Laboratories, Grand Island, NY, USA)](https://www.google.com/url?sa=t&rct=j&q=&esrc=s&source=web&cd=1&ved=2ahUKEwjZsdmj_JPiAhWEKqYKHUqQBJEQFjAAegQIABAB&url=https%3A%2F%2Fwww.thermofisher.com%2Fkr%2Fko%2Fhome%2Flife-science%2Fcell-culture%2Fmammalian-cell-culture%2Fclassical-media%2Fdmem.html&usg=AOvVaw33kWj0T-WtGSMuB74D14rv) supplemented with 20% FBS and 1% penicillin-streptomycin. Twenty-four hours later, the supernatant medium was replaced with 10% FBS–RPMI containing 3.5 x 10^6^ leukemia cells pre-incubated for two hours at 37°C either in 10% FBS–RPMI or in the same medium containing 1 µM LY2510924; the cells were then seeded on top of the stromal cells. Twenty-four hours later, quizartinib solution (4 ml with 2×concentration) was added and incubated with the cells at 37°C. Four hour later, the cells were lifted and pelleted. Leukemic cells were lysed and analyzed by immunoblotting for FLT3, STAT5, ERK, S6K, AKT, and GAPDH. We had to stain 11 proteins, including phospho-proteins and total proteins, for 12 samples, which should be tested at the same time to demonstrate precisely different effects of each group. Considering the number of proteins, we transversely cut the membrane to stain each protein at the same time. We assembled each membrane for proteins and developed whole blot image just prior to getting images for each band to prove the veracity (Supplementary Figure S5). Antibodies to AKT and ERK were obtained from Cell Signaling (Cell Signaling, Bedford, MA, USA). Each Western blot assay was performed in triplicate with stromal cells, and the same pattern was observed.

***AML mouse models***

MOLM-14/Luc/GFP cells were intravenously injected into NOD/SCID/IL-2rγnull (NSG) mice (The Jackson Laboratory, Bar Harbor, ME, USA) through tail vein at a concentration of 1×10^6^ cells/mouse to investigate the *in vivo* effects of treatment with LY2510924 with or without quizartinib. Bioluminescence imaging was used to monitor tumor burden. Briefly, mice were anesthetized and imaged noninvasively with an *in vivo* imaging system (Optical in vivo Imaging System-IVIS Lumina XRMS ; PerkinElmer, Waltham, MA, USA) after injection with luciferase substrate coelenterazine (Biotium, Heyward, CA, USA). Total body bioluminescence was quantified in a whole body of each mouse. After confirming leukemia engraftment by bioluminescence imaging, mice were randomized into treatment groups. Treatments consisted of LY2510924 (2.5 mg/kg subcutaneously, daily for 21 days) alone or together with a combination of quizartinib (2.5 mg/kg daily oral). The quizartinib was administered three hours after LY2510924 administration. To examine the LY2510924-induced mobilization of leukemic cells, peripheral blood was collected by facial blood collection before, three hours after, and 24 hours after LY2510924 administration, and the mobilized cells were detected by flow cytometry. The three mice for each group were euthanized to examine the extent of leukemic infiltration of different organs and femurs. Tissues were fixed in formalin, embedded in paraffin, and sectioned for immunohistochemical staining with anti-human CD45 antibody (abcam, Cambridge, UK). Remained mice for each group were monitored for survival.

***RNA sequencing and PCR***

For total RNA extraction, we used the miRNeasy Mini kit (Qiagen, Hilden, Germany) according to the manufacturer’s recommendation. The total mRNA was converted into cDNA library using TruSeq RNA Access Library Preparation Kit v2 (Illumina, San Diego, CA, USA). After polymerase chain reaction (PCR) amplification, the final product was assessed with a TapeStation (Agilent, Santa Clara, CA, USA) and sequenced on the Illumina NovaSeq 6000 platform (Illumina, San Diego, CA, USA) with 101-bp paired-end reads. Acquisition and processing of the sequencing data were performed as previously described.[^2^](#_ENREF_1) In brief, raw sequence data were first filtered and trimmed using Trimmomatic software.^3^ The sequencing reads were then mapped onto the human reference genome (GRCh38/hg38) using the STAR aligner v2.5.3a.^4^ Gene-level quantification of expression was performed with HTSeq v0.9.0,^5^ according to the Ensembl transcript annotation (Homo_sapiens_GRCh38.91 version). A differentially-expressed gene (DEG) analysis was performed for gene sets with expression by the use of edgeR R package.^6^ DEGs were defined by a false discovery rate of < 0.05 and a log2 fold change of > 1.

***Target specific quantitative reverse transcription PCR***

Reverse transcription was performed using oligo-(dT) primer and SuperScript® III reverse transcriptase (Thermo Fisher Scientific, Waltham, MA, USA). Details including primer information for targets and the endogenous control locus are available in Supplementary Table S2. Quantitative reverse transcription PCR (qRT-PCR) was performed using the ViiA7 real-time PCR system (Thermo Fisher Scientific, Waltham, MA, USA). In brief, 10 μl of reaction mixture contained cDNA, THUNDERBIRD™ SYBR qPCR Mix (TOYOBO, Osaka, Japan), 1× ROX, and 6 pmole of each primer. Thermal cycling conditions consisted of one cycle of one minute at 95°C followed by 40 cycles of five seconds at 95°C, 10 sec at 61°C, and 20 sec at 72°C. All qPCR experiments were carried out in triplicate and the expression level of each mRNA target was defined as ΔΔCt method as described elsewhere.^7^

***Statistical analyses***

In general, the results are expressed as the mean ± standard deviation (SD) of triplicate experiments or the mean ± standard error of the mean (SEM) for triplicate independent experiments. The Student’s paired t-test was used to compare differences between the groups. For the *in vivo* mouse experiments, the overall survival and mean group survival times were estimated by the Kaplan-Meier method and compared with the log-rank test. Differences of P ≤ 0.05 were considered statistically significant.

***References***

1. Schallmoser K, Strunk D. Preparation of pooled human platelet lysate (pHPL) as an efficient supplement for animal serum-free human stem cell cultures. J Vis Exp. 2009;(32):1523.

2. Jung SH, Kim MS, Jung CK, et al. Mutational burdens and evolutionary ages of thyroid follicular adenoma are comparable to those of follicular carcinoma. *Oncotarget.* 2016;7(43)**:**69638-69648.

3. Bolger AM, Lohse M, Usadel B. Trimmomatic: a flexible trimmer for Illumina sequence data. *Bioinformatics.* 2014;30(15)**:**2114-2120.

4. Dobin A, Davis CA, Schlesinger F, et al. STAR: ultrafast universal RNA-seq aligner. *Bioinformatics.* 2013;29(1)**:**15-21.

5. Anders S, Pyl PT, Huber W. HTSeq--a Python framework to work with high-throughput sequencing data. *Bioinformatics.* 2015;31(2)**:**166-169.

6. Robinson MD, McCarthy DJ, Smyth GK. edgeR: a Bioconductor package for differential expression analysis of digital gene expression data. *Bioinformatics.* 2010;26(1)**:**139-140.

7. Jung SH, Lee SE, Lee M, et al. Circulating microRNA expressions can predict the outcome of lenalidomide plus low-dose dexamethasone treatment in patients with refractory/relapsed multiple myeloma. *Haematologica.* 2017;102(11)**:**e456-e459.
